# Supplementary material for: Sequence-based genetic mapping of Cynodon dactylon Pers. reveals new insights into genome evolution in Poaceae
Source: Commun Biol. 2020 Jul 9;3:358. doi: 10.1038/s42003-020-1086-y (PMC7347563; doi:10.1038/s42003-020-1086-y)
Supplement: Supplementary file 1 — Supplementary Information [file 42003_2020_1086_MOESM1_ESM.pdf]

A.

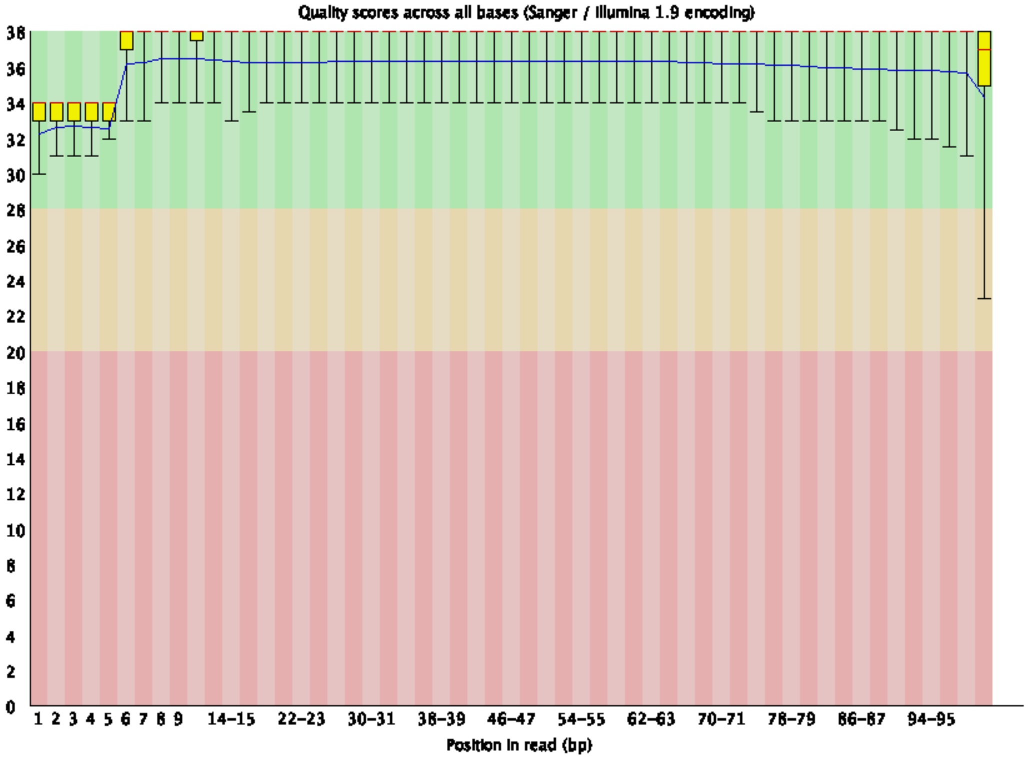
Quality scores across all bases (Sanger / Illumina 1.9 encoding)
38
36
34
32
30
28
26
24
22
20
18
16
14
12
10
8
6
4
2
0
1 2 3 4 5 6 7 8 9 14-15 22-23 30-31 38-39 46-47 54-55 62-63 70-71 78-79 86-87 94-95
Position in read (bp)

B.

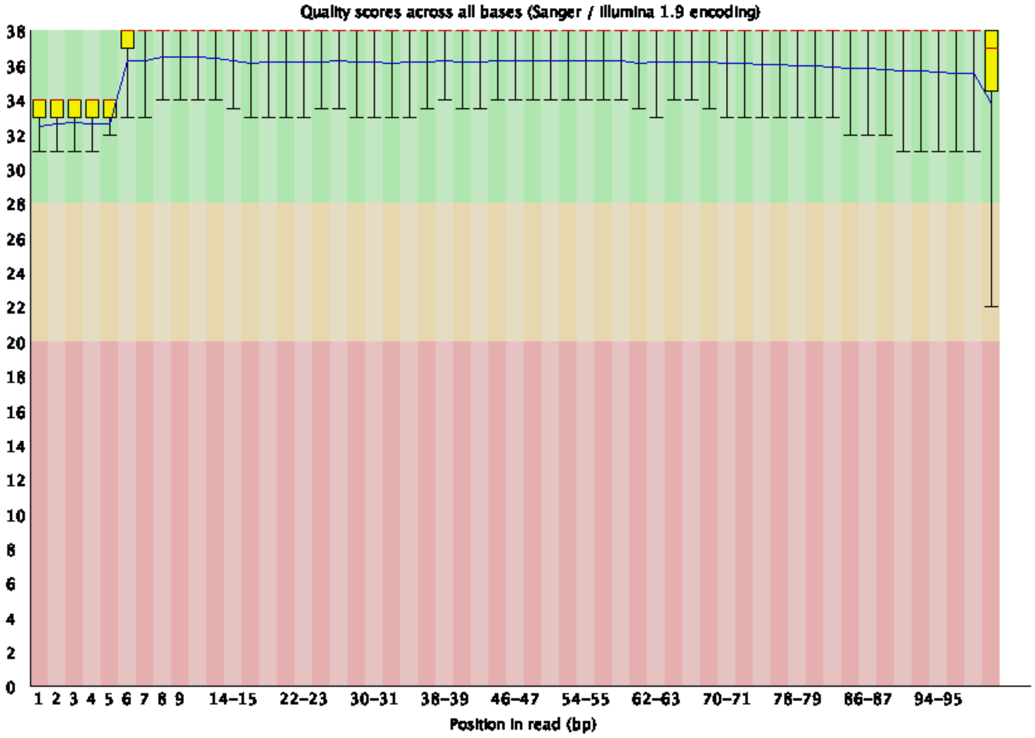
Quality scores across all bases (Sanger / Illumina 1.9 encoding)
38
36
34
32
30
28
26
24
22
20
18
16
14
12
10
8
6
4
2
0
1 2 3 4 5 6 7 8 9 14-15 22-23 30-31 38-39 46-47 54-55 62-63 70-71 78-79 86-87 94-95
Position in read (bp)

C.

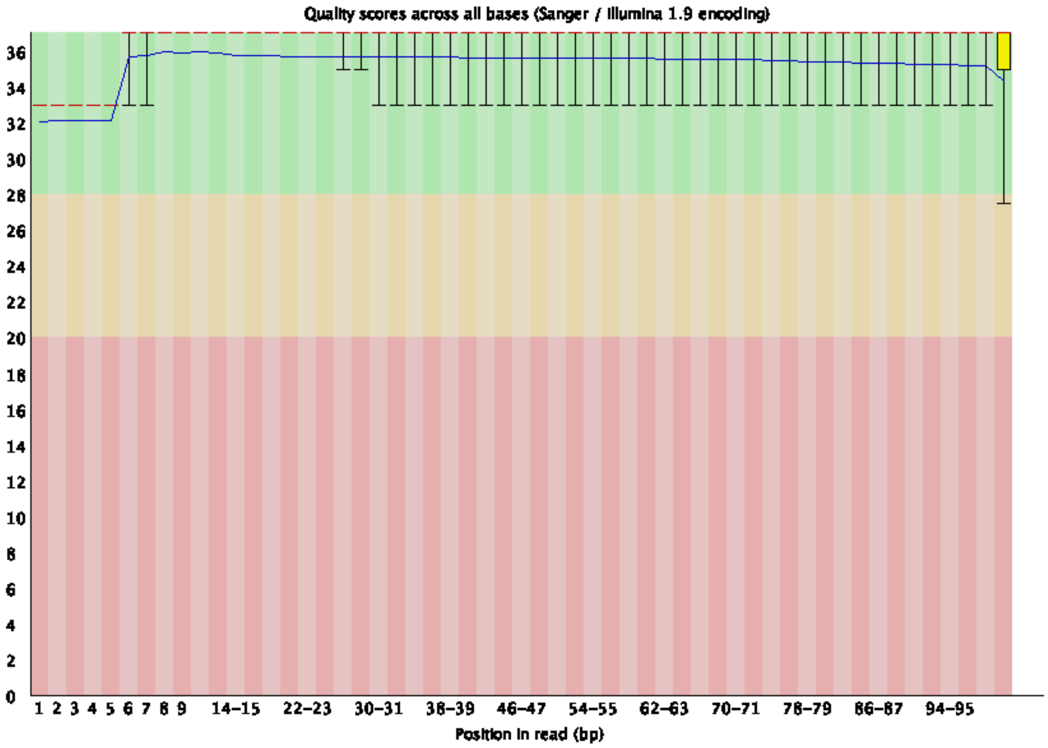

Supplementary Fig. 1. Base quality distribution of raw sequencing data in the common bermudagrass (*Cynodon dactylon*) population. A total of three libraries (A-C) were sequenced on an Illumina HiSeq2500 platform.

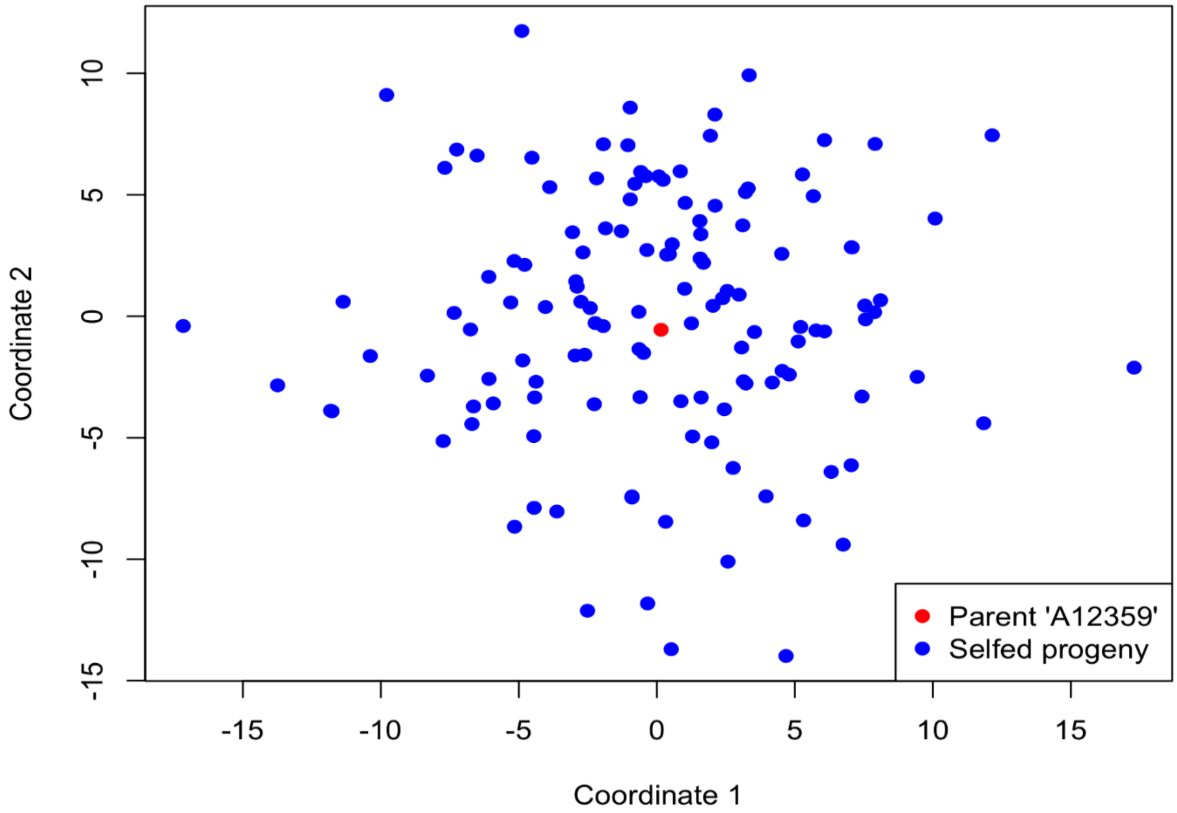

Supplementary Fig. 2. Two-dimensional plot of the principal coordinate analysis (PCoA) of 130 selfed progeny and their parent ‘A12359’ based on 3,544 markers. Red dot represents the parent, and blue dots represent the selfed progeny.

A

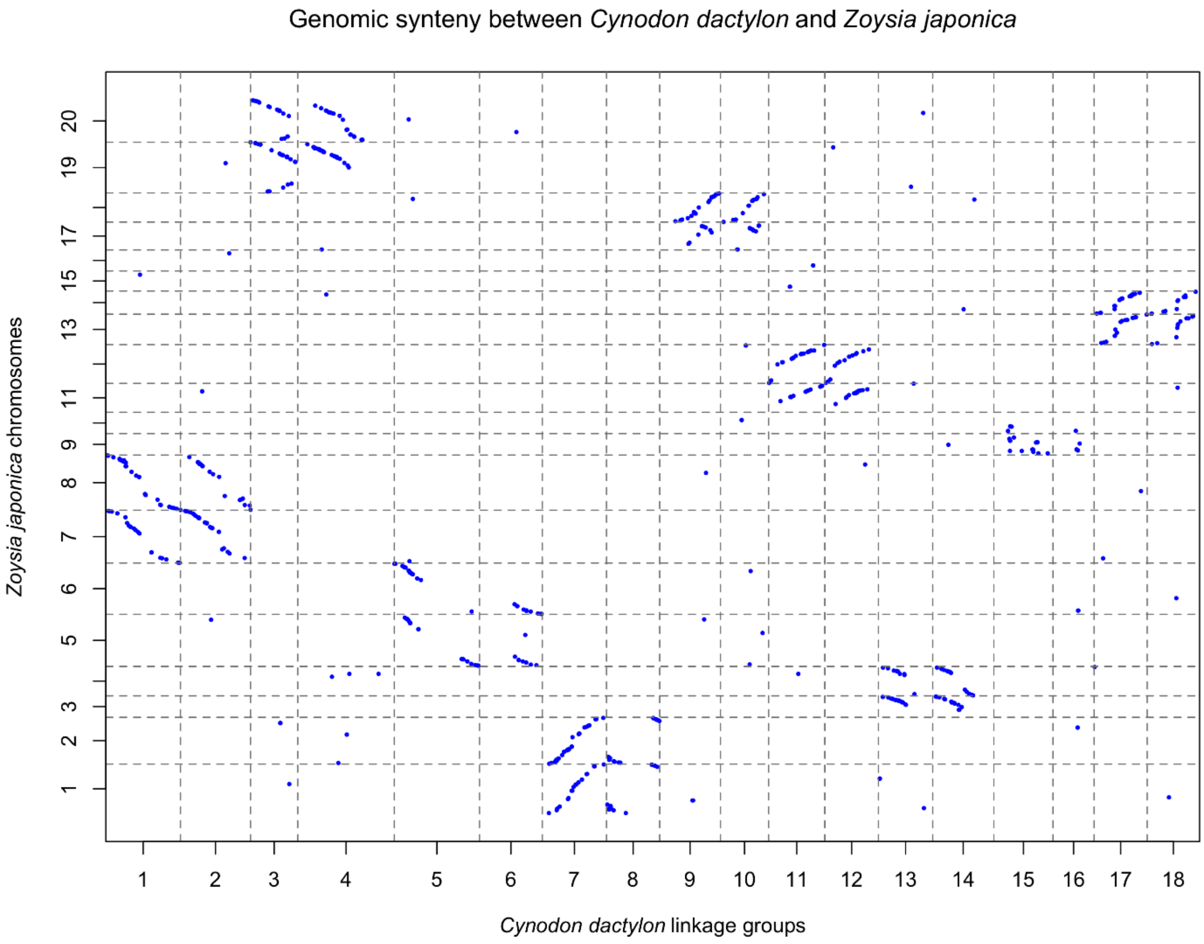

Genomic synteny between *Cynodon dactylon* and *Zoysia japonica*
Zoysia japonica chromosomes
1
2
3
5
6
7
8
9
11
13
15
17
19
20
Cynodon dactylon linkage groups
1
2
3
4
5
6
7
8
9
10
11
12
13
14
15
16
17
18

B

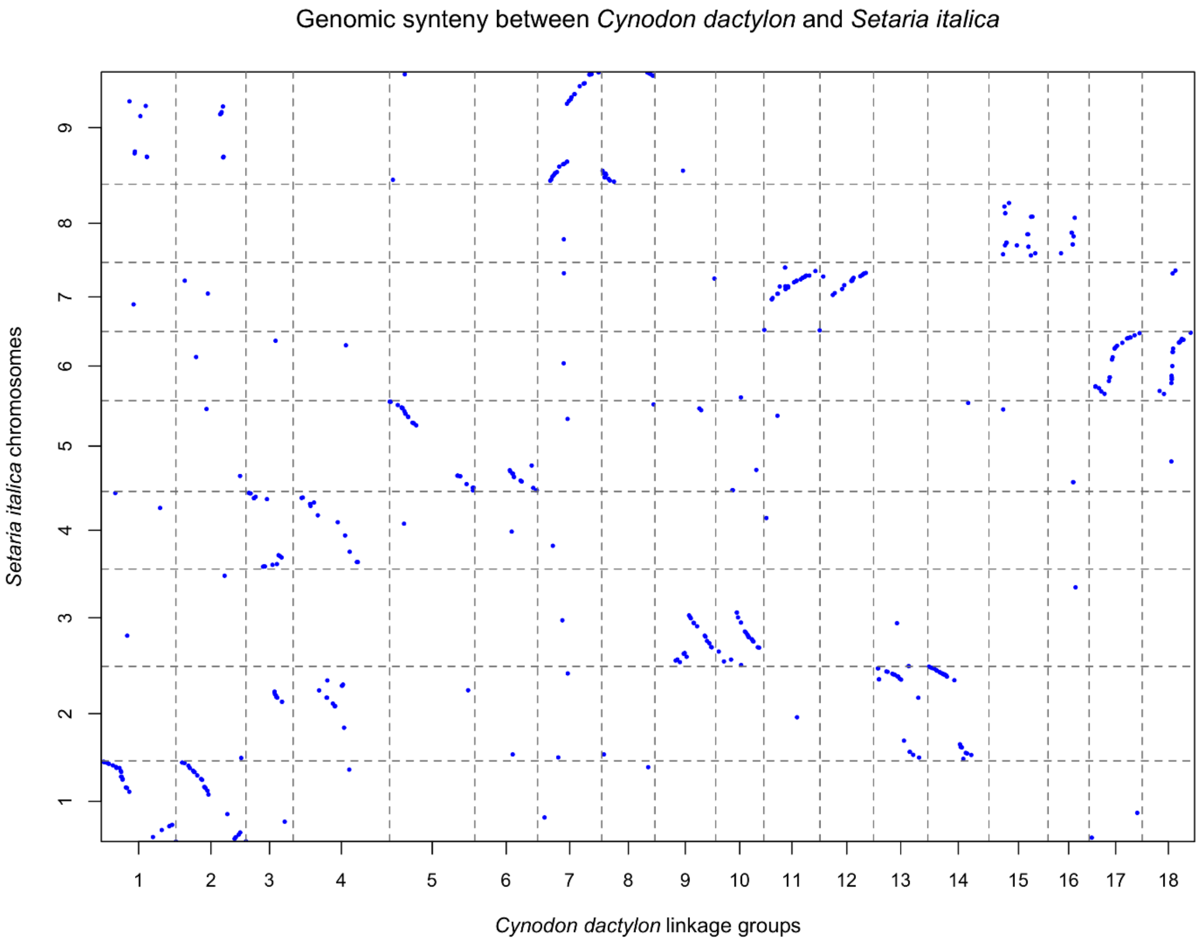

Genomic synteny between *Cynodon dactylon* and *Setaria italica*
Setaria italica chromosomes
1
2
3
4
5
6
7
8
9
Cynodon dactylon linkage groups
1
2
3
4
5
6
7
8
9
10
11
12
13
14
15
16
17
18

C

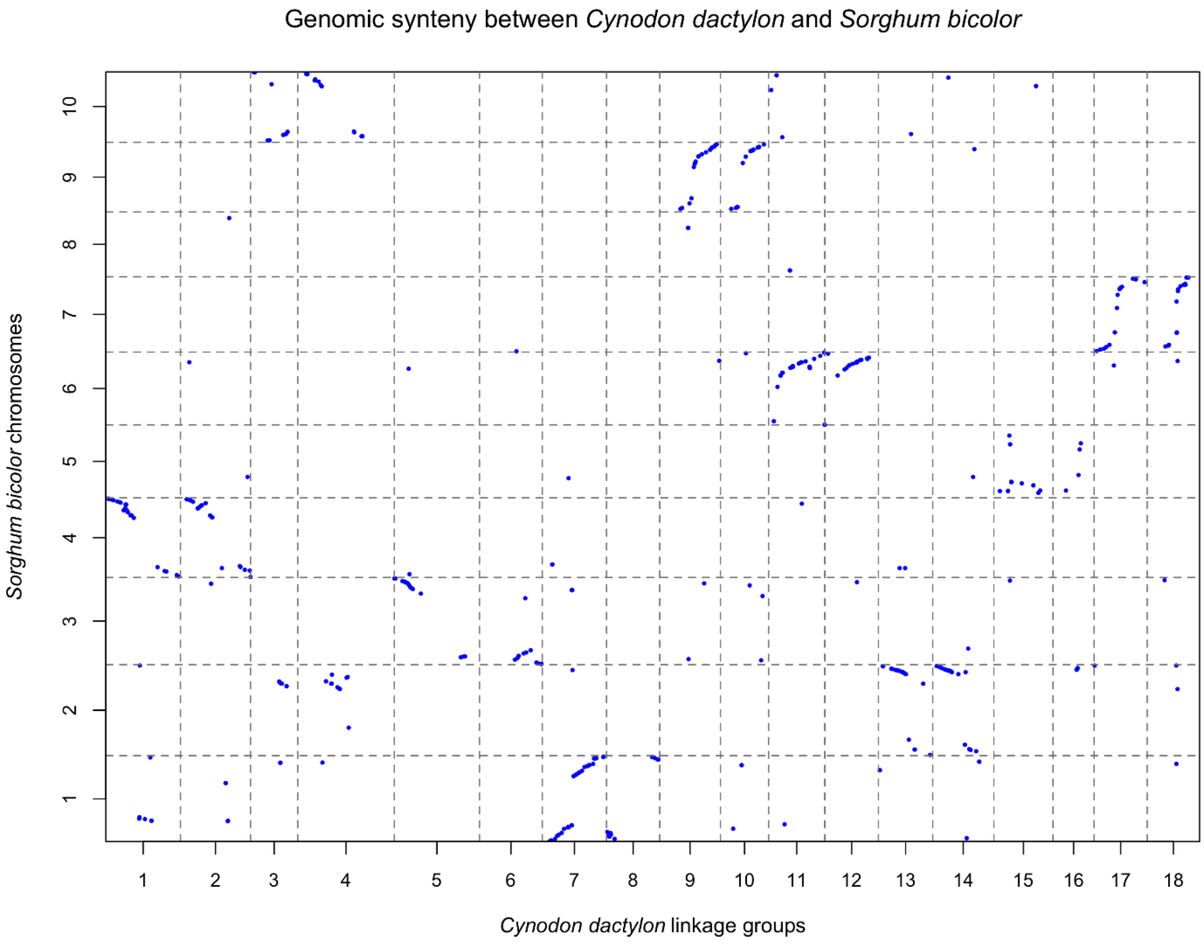
Genomic synteny between *Cynodon dactylon* and *Sorghum bicolor*
Sorghum bicolor chromosomes
1
2
3
4
5
6
7
8
9
10
Cynodon dactylon linkage groups
1
2
3
4
5
6
7
8
9
10
11
12
13
14
15
16
17
18

D

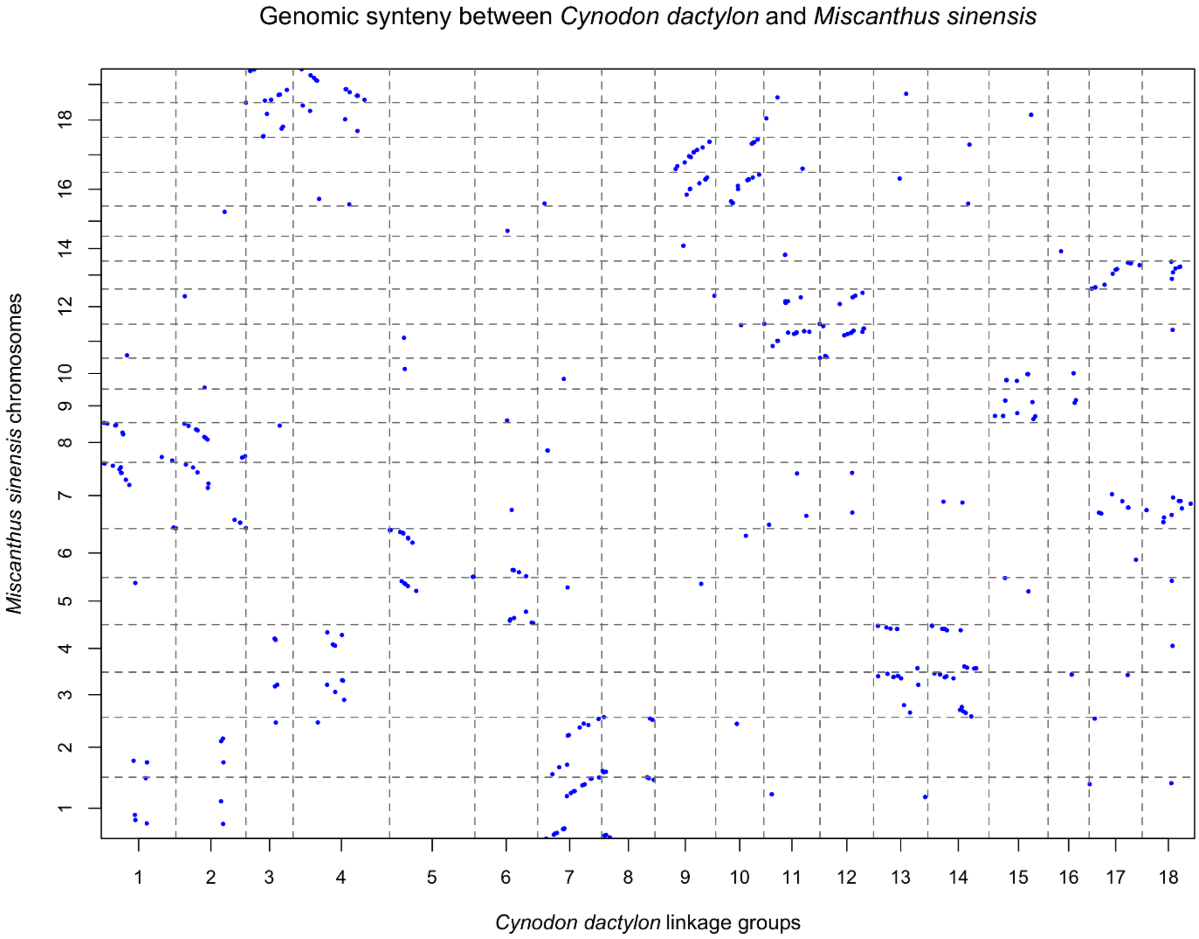

Genomic synteny between *Cynodon dactylon* and *Miscanthus sinensis*
Miscanthus sinensis chromosomes
1
2
3
4
5
6
7
8
9
10
12
14
16
18
1
2
3
4
5
6
7
8
9
10
11
12
13
14
15
16
17
18
Cynodon dactylon linkage groups

E

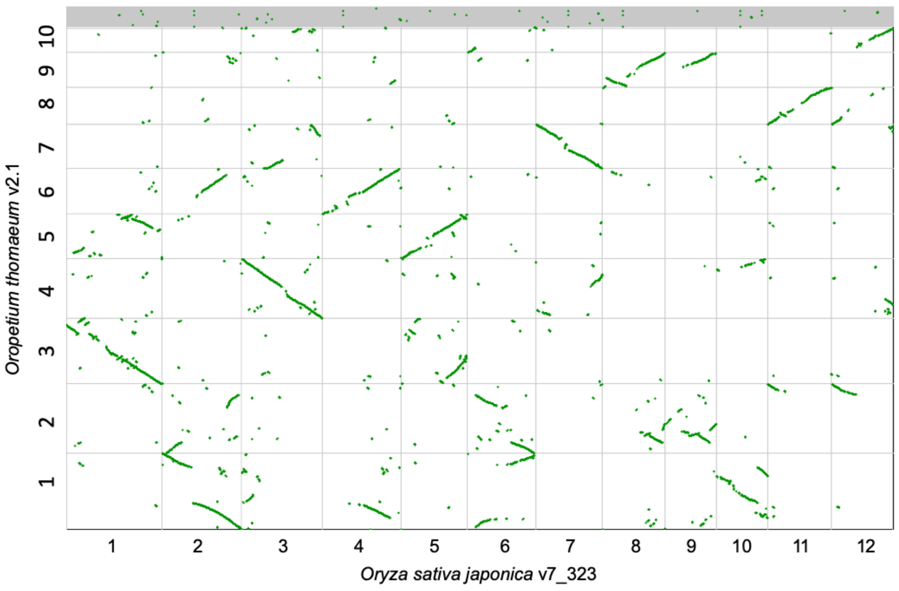

Supplementary Fig. 3. Genomic synteny plots between (A) *Cynodon dactylon* linkage groups (LGs) and *Zoysia japonica* chromosomes, (B) *C. dactylon* LGs and *Setaria italica* chromosomes, (C) *C. dactylon* LGs and *Sorghum bicolor* chromosomes, (D) *C. dactylon* LGs and *Miscanthus sinensis* chromosomes, and (E) *Oropetium thomaeum* and *Oryza sativa* chromosomes.
