# Supplementary material for: Sequence-based genetic mapping of Cynodon dactylon Pers. reveals new insights into genome evolution in Poaceae
Source: Commun Biol. 2020 Jul 9;3:358. doi: 10.1038/s42003-020-1086-y (PMC7347563; doi:10.1038/s42003-020-1086-y)
Supplement: Supplementary file 2 — Reporting Summary [file 42003_2020_1086_MOESM2_ESM.pdf]

nature research

Corresponding author(s): Yanqi Wu

Last updated by author(s): Jun 8, 2020

Reporting Summary

Nature Research wishes to improve the reproducibility of the work that we publish. This form provides structure for consistency and transparency in reporting. For further information on Nature Research policies, see Authors & Referees and the Editorial Policy Checklist.

Statistics

For all statistical analyses, confirm that the following items are present in the figure legend, table legend, main text, or Methods section.

n/a	Confirmed	
☐	☒	The exact sample size (*n*) for each experimental group/condition, given as a discrete number and unit of measurement
☐	☒	A statement on whether measurements were taken from distinct samples or whether the same sample was measured repeatedly
☐	☒	The statistical test(s) used AND whether they are one- or two-sided<br>*Only common tests should be described solely by name; describe more complex techniques in the Methods section.*
☒	☐	A description of all covariates tested
☒	☐	A description of any assumptions or corrections, such as tests of normality and adjustment for multiple comparisons
☒	☐	A full description of the statistical parameters including central tendency (e.g. means) or other basic estimates (e.g. regression coefficient) AND variation (e.g. standard deviation) or associated estimates of uncertainty (e.g. confidence intervals)
☒	☐	For null hypothesis testing, the test statistic (e.g. *F*, *t*, *r*) with confidence intervals, effect sizes, degrees of freedom and *P* value noted<br>*Give P values as exact values whenever suitable.*
☒	☐	For Bayesian analysis, information on the choice of priors and Markov chain Monte Carlo settings
☒	☐	For hierarchical and complex designs, identification of the appropriate level for tests and full reporting of outcomes
☒	☐	Estimates of effect sizes (e.g. Cohen's *d*, Pearson's *r*), indicating how they were calculated

Our web collection on statistics for biologists contains articles on many of the points above.

Software and code

Policy information about availability of computer code

Data collection	Sequence reads were trimmed to 64-bp in length, and to call SNPs by using the UNEAK pipeline in TASSEL
Data analysis	SNP filtering and comparative analysis were carried out with R code, which is given as "Bermudagrass_GBS_OSU" on GitHub. Mapchart input file is given in supplementary Table 3. Linkage map was constructed with JoinMap 5.

For manuscripts utilizing custom algorithms or software that are central to the research but not yet described in published literature, software must be made available to editors/reviewers. We strongly encourage code deposition in a community repository (e.g. GitHub). See the Nature Research guidelines for submitting code & software for further information.

Data

Policy information about availability of data

All manuscripts must include a data availability statement. This statement should provide the following information, where applicable:

- Accession codes, unique identifiers, or web links for publicly available datasets
- A list of figures that have associated raw data
- A description of any restrictions on data availability

All the relevant data and sequences of Tables 1, 2 and Fig. 1 are in the supplementary Table 1, Figs. 2-4 are in the supplementary Table 2 and 3.

Field-specific reporting

Please select the one below that is the best fit for your research. If you are not sure, read the appropriate sections before making your selection.

☒ Life sciences ☐ Behavioural & social sciences ☐ Ecological, evolutionary & environmental sciences

Life sciences study design

All studies must disclose on these points even when the disclosure is negative.

Sample size	130 progeny from 1 parent
Data exclusions	Initially, SNPs were obtained with loose SNP calling criteria (minor allele frequency >= 0.01, missing data per site < 90%). In order to reduce heterozygotes undercalling (i.e., incorrectly call a heterozygous genotype as homozygote due to low coverage), a minimum of six reads per SNP was imposed to call heterozygotes with a theoretical accuracy of 96.88% by assuming a binomial distribution of reads at two alleles each locus. Genotype data with ≤ five reads were converted to missing data. Then SNPs with missing rate no more than 10% were retained.
Replication	The genotyping by sequencing (GBS) for each of the 130 S1 progenies was sequenced with two replicates and the parent was sequenced with 14 replicates.
Randomization	130 randomly selected individuals from the S1 population were implemented for GBS and genetic map construction.
Blinding	Not relevant

Reporting for specific materials, systems and methods

We require information from authors about some types of materials, experimental systems and methods used in many studies. Here, indicate whether each material, system or method listed is relevant to your study. If you are not sure if a list item applies to your research, read the appropriate section before selecting a response.

Materials & experimental systems

n/a	Involved in the study
☒	☐ Antibodies
☒	☐ Eukaryotic cell lines
☒	☐ Palaeontology
☒	☐ Animals and other organisms
☒	☐ Human research participants
☒	☐ Clinical data

Methods

n/a	Involved in the study
☒	☐ ChIP-seq
☒	☐ Flow cytometry
☒	☐ MRI-based neuroimaging
