# Supplementary material for: Sequence-based genetic mapping of Cynodon dactylon Pers. reveals new insights into genome evolution in Poaceae
Source: Commun Biol. 2020 Jul 9;3:358. doi: 10.1038/s42003-020-1086-y (PMC7347563; doi:10.1038/s42003-020-1086-y)
Supplement: Supplementary file 7 — Description of Additional Supplementary Files [file 42003_2020_1086_MOESM7_ESM.docx]

Supplementary Data 1. Comparative genomics database of markers in the *Cynodon dactylon* genetic map.

Supplementary Data 2. Genetic map of 320 SNVs markers that explained 10-to-9 chromosome reduction.

Supplementary Data 3. Input file for MapChart for segregation distorted markers on linkage groups (LGs) 1 to 18, 3-1, 4-1 5-1 and 6-1.
